# Parental Educational Attainment and Chronic Medical Conditions among American Youth; Minorities’ Diminished Returns

**DOI:** 10.3390/children6090096

**Published:** 2019-08-26

**Authors:** Shervin Assari, Mohsen Bazargan, Cleopatra H. Caldwell

**Affiliations:** 1 Department of Family Medicine, Charles R Drew University of Medicine and Science, Los Angeles, CA 90059, USA; 2 Department of Family Medicine, University of California Los Angeles, Los Angeles, CA 90095, USA; 3 Department of Psychology, University of California, Los Angeles (UCLA), Los Angeles, CA 90095, USA; 4 Department of Health Behavior and Health Education, School of Public Health, University of Michigan, Ann Arbor, MI 48104, USA

**Keywords:** population groups, ethnicity, ethnic groups, race, Whites, Hispanics, Latino, Blacks, African-Americans, socioeconomic position, socioeconomic status, education, chronic diseases

## Abstract

Background: Parental educational attainment is protective against chronic medical conditions (CMCs). According to the minorities’ diminished returns (MDRs) theory, however, the health effects of socioeconomic status (SES) indicators are smaller for socially marginalized groups such as racial and ethnic minorities rather than Whites. Aims: To explore racial and ethnic differences in the effect of parental educational attainment on CMCs in a nationally representative sample of American youth. Methods: In this cross-sectional study, we used baseline data of 10,701 12–17 years old youth in the Population Assessment of Tobacco and Health (PATH; 2013). Parental educational attainment was the independent variable. The dependent variable was the number of CMCs in youth. Age, gender, and family structure were covariates. Race and ethnicity were the focal moderators. Linear and multinomial regression were applied to analyze the data. Results: Overall, higher parental educational attainment was associated with a lower number of CMCs. Race and ethnicity, however, showed significant interactions with parental educational attainment on a number of CMCs as well as 2+ CMCs, suggesting that the effect of parenting educational attainment on CMCs is significantly smaller for Black and Hispanic than White youth. Conclusions: In the United States, race and ethnicity alter the health gains that are expected to follow parental educational attainment. While White youth who are from highly educated families are most healthy, Black and Hispanic youth from highly educated families remain at higher risk for CMCs. That means, while the most socially privileged group, Whites, gain the most health from their parental education, Blacks and Hispanics, the least privileged groups, gain the least. The result is a disproportionately high number of CMCs in middle-class Blacks and Hispanics. Economic, social, public, and health policy makers should be aware that health disparities are not all due to lower SES of the disadvantaged group but also diminished returns of SES resources for them. Youth physical health disparities due to race and ethnicity exist across all SES levels.

## 1. Background

Compared to White youth, Black youth have a higher number of chronic medical conditions (CMCs) in the US [1,2]. For example, Black youth are at an increased risk of obesity [3,4,5], asthma [6], diabetes [7], and hypertension [8,9,10,11,12,13,14] compared to their White counterparts. Disparities in CMCs are among the main contributors to racial and ethnic health disparities and extends to disparities in mortality [15]. Inequality in CMCs in youth also is a gateway to the very persistent racial and ethnic disparities in the morbidity and mortality later in life [1].

Socioeconomic status (SES) has protective effects against health risks such as obesity [16,17,18] and CMCs in youth [19,20] and adults [21,22]. As a result, some of the disparities in CMCs are attributed to the SES gap across race and ethnic groups [17,23,24,25]. Among various SES indicators, parental educational attainment is among the main protective factors against CMCs among youth [26,27,28]. This is in part because parental educational attainment reflects social status and upward social mobility as well as income [29,30]. Through a wide range of mechanisms such as availability of materialistic resources, parenting, health behaviors, and self-care, parental educational attainment is a major determinant of health of the children [31,32,33].

Minorities’ diminished returns (MDRs) [34,35] refer to “less than expected” protective effects of SES indicators on health outcomes for the members of socially marginalized groups compared to the socially privileged group [17,24,33]. A large body of literature has documented MDRs of various SES indicators for Blacks and Hispanics compared to Whites. The MDRs suggest that: (a) Not all are due to SES gaps across racial and ethnic groups but also because of the differential health gains that follow SES resources such as parental educational attainment for marginalized populations such as Blacks and Hispanics, and (b) the relative racial and ethnic gap in health widens, instead of shrinking, as SES increases [17,24,33], and (c) there is a need to study racial and ethnic health disparities across all SES levels, not just among low SES groups [34,35].

We conducted this study to test whether race and ethnicity modify the effect of parental educational attainment on the number of CMCs among youth in the US. In line with the MDRs literature [17,24,33], we hypothesized that the protective effect of parental educational attainment would be significantly smaller for Black and Hispanic youth than White youth. As a result of unequal life circumstances, we expect to observe MDRs for the effects of parental educational attainment on the number of CMCs for Black and Hispanic than White youth. The unique contribution of this study is for the first time to extend the literature on MDRs on CMCs from adults [36] to youth. In addition, although MDRs are shown for individual CMCs such as depression [37], obesity [33], asthma [38], and attention deficit hyperactivity disorder (ADHD) [39] for Black youth, no previous studies have tested MDRs for CMCs in Hispanic youth.

## 2. Methods

### 2.1. Design and Settings

This is a secondary analysis of wave 1 of the Population Assessment of Tobacco and Health (PATH) youth data. Funded by the NIH and FDA, PATH is a state-of-the art study on health problems such as tobacco use, substance use, and related behavioral issues among American adolescents and adults. Overall, PATH has enrolled about 49,000 people who were 12 years or older at baseline. From this sample, about 14,000 were youth (12–17 years old). Wave 1 data were collected between 2013 and 2014. Although PATH also has recruited adults, this analysis is only focused on youth. We used publicly available PATH data set, which was downloaded from the ICPSR website. We merged data sets using the unique identifiers that are present in the Master Linkage file. As we were interested in the association between SES and number of CMCs, we decided to use the cross-sectional data and limited our variables to the wave 1 of the PATH study.

### 2.2. Sample and Sampling

The PATH study’s population of interest in Wave 1 was the civilian, non-institutionalized US population 12 years of age and older individuals in the US. The PATH study used a four-stage stratified area probability sample design. At the first stage, a stratified sample of geographical primary sampling units (PSUs, *n* = 156) was selected. These PSUs were either a county or a group of counties. The second stage formed and sampled smaller geographical segments in each PSU. The third-stage sampled residential addresses, using the US postal service computerized delivery sequence files. The fourth stage was the selection of one person from each sampled household. 

### 2.3. Analytical Sample

The current analysis is limited to youth who had valid data on age, gender, parental education, marital status, race, ethnicity, and number of CMCs. Our final analytical sample was 10,701 (8678 White and 2023 Black) youth. The major drop in the sample was not due to missing samples but due to the exclusion of participants who belonged to any race other than White and Black. Thus, participants were excluded if they were Asian American, Native American, multi-racial, or had marked other races.

### 2.4. Study Variables

The study variables include demographic factors (age and gender), race, ethnicity, SES indicators (parental educational attainment), marital status of the parents (family structure), and number of CMCs. 

*Demographic Factors:* Age was a dichotomous variable as below: (1) 12 to 15 years old, and (2) 16 to 18 years old.

*Race and Ethnicity:* Race was self-identified and operationalized as a dichotomous variable: Black versus White. Ethnicity was also self-identified and operationalized as a dichotomous variable: Non-Hispanic versus Hispanic. 

*Socioeconomic Status:* Parental educational attainment was a five-level variable as below: (1) Less than high school, (2) high school graduate or equivalent, (3) some college including no degree or associates degree, (4) bachelor’s degree, and (5) advanced degree. This variable was treated as a continuous measure ranging from 1 to 5, with a higher score indicating higher family SES.

*Marital Status:* The parent who was interviewed reported the marital status (family structure) of the household. This variable was operationalized as a dichotomous variable (0 not married, 1 married).

*Chronic Medical Condition (CMCs):* In this study, the number of CMCs was measured by asking parents to report about the presence of 11 CMCs in their child. Parents were asked by the interviewer if a physician had ever told them that their child has any of these conditions: hypertension (HTN), high cholesterol, asthma, bronchitis/pneumonia, attention deficit hyperactivity disorder (ADHD), a dental problem, and diabetes mellitus (DM). Parent-reports provide valid information regarding CMCs, although some bias in this approach is to be expected. We had two outcomes: Number of CMCs for the linear regression and 0, 1, and 2+ CMCs, for our multinomial regression model.

### 2.5. Conceptual Model

Built on the MDRs, our study was mainly focused on the interaction between race and SES. As a result, the main predictor of interest is SES (parental educational attainment). The outcome of interest was the number of CMCs. We expected that the high family SES (parental educational attainment) reduced the number of CMCs, however, we expected this effect to be smaller in Black and Hispanics compared to White youth. 

### 2.6. Data Analytical Plan

We analyzed the data using SPSS 23.0 (IBM Corporation, Armonk, NY, USA). PATH is a survey and involves weights. Using SPSS, we applied Taylor series linearization and re-estimated the standard errors, using sampling weights. Thus, the results are representative to the US population.

To analyze our data, first, we examined the distribution of our categorical and continuous variables to rule out multi-collinearity between independent variables. For the univariate analysis, we used frequency tables as well as means (SD). For bivariate analysis, we used a Pearson correlation test. For the multivariable analysis, we applied linear regression models as well as multinomial regression models. First, we excluded multicollinearity between the study variables. We also tested the distribution of the errors before we ran our linear regression model. We also ran a polynomial regression model. We ran models in the pooled sample. Still, we did not solely rely on the results of linear regression model that has more assumptions. We replicated our findings of linear regression using multinomial regression. From linear regressions, we reported b, SE, 95% CI, and *p* values. From multinomial regressions, we reported b, SE, ORs, 95% CI, and *p* values. We did not impute the missing data. No variable was missing in more than 5%, thus the completeness rate for all study variables were more than 95%.

### 2.7. Ethics

All youth who participated in the PATH study provided assent. All their parents/guardians/caregivers provided a written informed consent. The PATH study protocol was approved by the Westat institutional review board.

## 3. Results

### 3.1. Descriptive Statistics

This study included 10,701 American youth. Table 1 shows descriptive statistics of the overall sample.

In total, there were 52.5% (*n* = 5578) youth with no CMC, 33.7% (*n* = 3580) with one CMC, and 13.7% (*n* = 1458) had 3+ CMCs. Appendix A shows the percentage of individuals with various number of CMCs based on the intersection of age and ethnicity.

### 3.2. Bivariate Analysis

Race, ethnicity, age, gender, parental marital status, and parental educational attainment were correlated with number of CMCs. Race (Black) and age were positively correlated with number of CMCs, however, ethnicity (Hispanics), parental marital status (married), and high parental educational attainment were correlated with a lower number of CMCs. Gender (male) was also positively correlated with number of CMCs (Table 2).

### 3.3. Multivariable Models in the Pooled Sample

Table 3 presents the summary of the results of two linear regression models with parental educational attainment as the independent variable and number of CMCs as the dependent variable. Based on our multivariate models, Hispanics (coded = 1) had less (not more) CMCs. We also observed that being Black was not significant in our non-interaction model after adjusting for the education levels. Based on *Model 1*, high educational attainment was associated with lower number of CMCs. *Model 2* showed significant interactions between race and ethnicity with parental educational attainment on number of CMCs, suggesting that high parental educational attainment had smaller protective effects on number of CMCs for Black and Hispanic youth than White youth (Table 3).

### 3.4. Multivariable Models in the Pooled Sample

Table 4 and Table 5 present the summary of the results of two multinomial regression models with parental educational attainment as the independent variable and 0, 1, and 2+ CMCs as the dependent variable. Both models were estimated in the overall sample. *Model 3* only entered the main effects of educational attainment, race, and ethnicity. *Model 4* also added two interaction terms between race and ethnicity with educational attainment.

Based on *Model 3*, high educational attainment was not associated with 1 CMC, however, it was associated with lower odds of 2+ CMCs. *Model 4* showed significant interactions between race and ethnicity with parental educational attainment on two CMCs, suggesting that high parental educational attainment had smaller protective effects against 2+ CMCs for Black and Hispanic than White youth (Table 4 and Table 5).

## 4. Discussion

The current study showed two findings. First, overall, higher parental educational attainment was associated with a lower number of CMCs in American youth. Second, race and ethnicity showed significant interactions with parental educational attainment suggesting that high parental educational attainment had a smaller protective effect against a high number of CMCs for Black and Hispanic youth than White youth.

Our first finding was in line with the extensive literature on fundamental causes [40,41,42], and social determinants of health (SDOH) [43,44,45]. A large body of research has shown that parental educational attainment is associated with better health and well-being [26,29,46,47] and also a lower risk of obesity [48]. We extended this literature to the number of CMCs.

Built on our previous work on MDRs that shows high SES Black and Hispanic youth remain at an increased risk of poor health outcomes such as obesity, CMCs, and depression, compared to high SES Whites [17,24,33]. A pattern that is seen for a wide range of SES indicators and health outcomes, for youth, adults, and older adults [49,50,51,52]. Similar patterns are reported for educational attainment [24,33], income [17,53], employment [54], and marital status [55] on obesity [24,33], chronic disease [39], depression [53], anxiety [55], self-rated health [56,57], and even mortality [54]. In several papers, smaller effects of SES indicators are shown for Blacks and Hispanics than Whites [34,35].

We argued that various aspects of structural racism, residential segregation, and labor market discrimination all contribute to MDRs of educational attainment on the number of CMCs [33]. There is a need to understand the role of an unsafe environment [58], food deserts [59,60,61,62], and the density and availability of unhealthy [63,64,65,66] and healthy [67] food choices and options across locations based on the intersection of race and SES. Scarce educational resources also reduce quality of education in in predominantly Black and Hispanic schools. Educational attainment has a smaller effect on increasing Blacks’ and Hispanics’ employment and enhancing their life conditions less compared to Whites [29,30,46,68]. For example, a larger proportion of Blacks with high educational attainment remain under poverty compared to Whites [68].

Conceptualizing race as a social [69,70,71] and political [72,73,74,75] rather than biological factor, we argue that MDRs are not because minority groups are inherently unable to efficiently transform their available resources to tangible health outcomes but it is the differential treatment by the society (racism) that marginalizes and stigmatizes racial minority groups [34,35]. Considerable theoretical and empirical work has discussed how political power [72,73,74,75] is unevenly distributed between Blacks and Whites.

The study also showed other results. From the multivariate models, it looks like being Hispanic has less (not more) CMCs. Being Black was also not significant in the main effect model that adjusted for education levels.

### 4.1. Implications

The results may have some implications for research, policy, and practice. First, we argued that public, economic, social, and health policy makers should not assume that SES indicators are equally protective against health problems across racial and ethnic groups, as high SES Blacks and Hispanics remain at high risk of CMCs. As new policies are being implemented, there is a need for “impact analysis” to track effects on the populations overall, and also sub-population differences in such effects. There is also a need to conduct further studies that can help us understand why youth from high SES families remain susceptible to CMCs. The results may contribute to the development of public policies that can eliminate or at least reduce the existing CMCs disparities in the US, which is a strategic priority for CDC, NIH, and other authorities. We argued that providing incentives for health in predominantly Black areas might be required. An enhancement to the quality of education and schooling in predominantly Black areas is also needed. Finally, there is a need to reduce racism and discrimination that limit translation of educational attainment to health outcomes. This is very important given public, social, and health policies are much more effective than individual-level interventions that overemphasize individual choices, preferences, and behaviors [76]. There is a need for policies at national and local levels that can reduce racial, ethnic, and SES health disparities that are caused by MDRs [31,33,35,50,51,52,55,56,77,78].

### 4.2. Limitations

This study had some limitations. First, given the cross-sectional design of our data, causal inferences were not plausible. Sample size was imbalanced across racial and ethnic groups, which is a common problem in national surveys. In addition, we did not have access to a wide range of SES indicators of the household such as income, employment, and wealth. Our measure of educational attainment was also not ideal. Having an advanced degree is quite broad. The impact of educational attainment presumably depends on the university quality/prestige. Such effect may also depend on where the degree is taken. Using geocoded data, future research may also consider the university and state level characteristics to the model. Future research may also model area level SES indicators such as racial composition, neighborhood SES, availability of resources such as park and green space, as well as risk factors such as fast foods. In addition, this study used a simplistic measure of CMCs, which was the parent report. Finally, we did not include area level SES indicators. Despite these limitations, this study contributes to the literature on race, SES, and multimorbidity.

### 4.3. Future Research

Various SES indicators such as parental educational attainment do not have magical effects and their effects are due to a wide range of processes and mechanisms. For example, a healthy diet, stress, exercise, and the social and physical environment may explain why parental educational attainment protect individuals’ health. Similarly, children of high SES families may have lower risk peers, attend better schools, have better teachers, and live in low stress environment. Parents with higher educational attainment may also receive higher levels of parenting and social support. All of these processes are potentially protective and may be diminished for non-White families. Therefore, there is a need to study the environmental and behavioral mediators of MDRs of higher SES for Black and Hispanic families. In addition, whether there are consistent or inconsistent MDRs across various CMC outcomes should be examined. Some specific types of CMCs may be more prone than others to MDRs of parental SES. That means, research should determine the type of CMCs that Blacks, Latinos, and Whites gain similarly from high parental educational attainment of the parents.

## 5. Conclusions

In the United States, race and ethnicity limit the health gains that follow the availability of SES indicators. While high parental education helps youths avoid CMCs, the most privileged group gains the most and the least privileged groups gain the least from such potential. As a result, we should expect additional CMCs in middle-class Blacks and Hispanics. Researchers should not take a minimalistic and over-simplistic approach and reduce the problem of racial and ethnic health disparities to low SES among Blacks and Hispanics. They should consider that disparities can be observed across all SES levels, and the SES gap is only one part of the story. As health disparities are also a problem with middle class Blacks, a real solution should also target other sections of Black and Hispanic communities that may not be low SES but still at an increased risk of poor health outcomes. Policy makers should also go beyond equalizing SES by addressing the barriers that are more common in the lives of Blacks and Hispanics. Such barriers may hinder non-Whites’ ability to turn their available SES resources to tangible health outcomes.

## Figures and Tables

**Table 1:** Descriptive statistics in the overall sample.

	*n*	*%*
Race		
White	8678	81.1
Black	2023	18.9
Ethnicity * ^a^		
Non-Hispanic	8179	77.8
Hispanic	2329	22.2
Age		
12–15	5474	51.2
16–17	5227	48.8
Gender		
Women	5143	48.2
Men	5531	51.8
Marital Status * ^a^		
Not Married	3870	36.2
Married	6817	63.8
CMCs * ^a^		
None	5578	52.5
One	3580	33.7
2+	1458	13.7
	*Mean*	*SD*
Educational Attainment (1–5) * ^b^	2.84	1.22
Number of CMCs * ^b^	0.65	0.81

* *p* < 0.01, ^a^ Chi square test, ^b^ independent samples *t* test.

**Table 2:** Bivariate correlations in the overall sample.

	1	2	3	4	5	6	7
1 Race (Black)	1.00	−0.14 **	0.00	0.00	−0.25 **	−0.19 **	0.12 *
2 Ethnicity (Hispanic)		1.00	0.00	−0.14 **	−0.14 **	−0.28 **	−0.12 *
3 Gender (Male)			1.00	0.01	0.00	0.11	0.19 **
4 Age (Years)				1.00	0.00	−0.11	0.12*
5 Marital Status (Married)					1.00	0.19 **	−0.08 **
6 Educational Attainment (Family SES; 1–5)						1.00	−0.13 **
7 Number of CMCs							1

* *p* < 0.05 ** *p* < 0.01. SES: Socioeconomic status. Pearson correlation test.

**Table 3:** Summary of linear regressions without and with interactions on the number of CMCs in the pooled sample.

	B	SE	Beta	95% CI		*p*
*Model 1: Main Effect*						
Race (Black)	−0.01	0.02	0.00	−0.05	0.03	0.677
Ethnicity (Hispanic)	−0.07	0.02	−0.03	−0.11	−0.03	0.001
Marital Status (Married)	−0.14	0.02	−0.08	−0.17	−0.10	0.000
Gender (Male)	0.15	0.02	0.09	0.12	0.18	0.000
Age (16–17)	0.03	0.02	0.02	0.00	0.06	0.032
Educational Attainment	−0.02	0.01	−0.02	−0.03	0.00	0.023
Constant	0.70	0.03		0.65	0.76	0.000
*Model 2: Interaction Effect*						
Race (Black)	−0.17	0.05	−0.08	−0.27	−0.07	0.001
Ethnicity (Hispanic)	−0.24	0.04	−0.12	−0.33	−0.16	0.000
Marital Status (Married)	−0.13	0.02	−0.08	−0.17	−0.10	0.000
Gender (Male)	0.15	0.02	0.09	0.12	0.18	0.000
Age (16–17)	0.03	0.02	0.02	0.00	0.06	0.037
Educational Attainment	−0.04	0.01	−0.06	−0.06	−0.03	0.000
Race × Educational Attainment	0.06	0.02	0.08	0.02	0.09	0.001
Ethnicity × Educational Attainment	0.07	0.02	0.09	0.04	0.10	0.000
Constant	0.78	0.03		0.72	0.84	0.000

Notes: Source Population Assessment of Tobacco and Health (PATH; 2013–2014). CI: Confidence interval; SE: Standard error.

**Table 4:** Summary of multinomial regression (Model 3) with only main effects on the number of Chronic Medical Conditions (CMCs) in the pooled sample.

	b	SE	OR	95% CI		*p*
Outcome: 1 CMC						
Race (Black)	−0.02	0.06	0.98	0.87	1.10	0.731
Ethnicity (Hispanic)	−0.19	0.06	0.83	0.74	0.92	0.001
Marital Status (Married)	−0.26	0.05	0.77	0.71	0.85	0.000
Gender (Male)	0.27	0.04	1.31	1.20	1.43	0.000
Age (16–17)	0.02	0.04	1.02	0.94	1.11	0.641
Educational Attainment	0.02	0.02	1.02	0.98	1.06	0.324
Constant	−0.43	0.08				0.000
Outcome: 2+ CMCs						
Race (Black)	−0.04	0.08	0.96	0.83	1.13	0.651
Ethnicity (Hispanic)	−0.21	0.08	0.81	0.70	0.95	0.007
Marital Status (Married)	−0.47	0.06	0.62	0.55	0.71	0.000
Gender (Male)	0.56	0.06	1.75	1.55	1.97	0.000
Age (16–17)	0.11	0.06	1.11	0.99	1.25	0.073
Educational Attainment	−0.06	0.03	0.94	0.89	0.99	0.015
Constant	−1.18	0.10				0.000

Notes: Source Population Assessment of Tobacco and Health (PATH; 2013–2014). CI: Confidence interval; SE: Standard error; OR: Odds ratio.

**Table 5:** Summary of multinomial regression (Model 4) with interactions on the number of Chronic Medical Conditions (CMCs) in the pooled sample.

	b	SE	OR	95% CI		*p*
Outcome: 1 CMC						
Race (Black)	−0.34	0.15	0.71	0.54	0.95	0.019
Ethnicity (Hispanic)	−0.47	0.13	0.62	0.49	0.80	0.000
Marital Status (Married)	−0.25	0.05	0.78	0.71	0.85	0.000
Gender (Male)	0.27	0.04	1.31	1.21	1.43	0.000
Age (16–17)	0.02	0.04	1.02	0.94	1.11	0.667
Educational Attainment	−0.03	0.02	0.97	0.93	1.02	0.248
Race × Educational Attainment	0.11	0.05	1.12	1.02	1.23	0.019
Ethnicity × Educational Attainment	0.11	0.05	1.12	1.02	1.22	0.015
Constant	−0.29	0.09				0.001
Outcome: 2+ CMCs						
Race (Black)	−0.60	0.19	0.55	0.38	0.81	0.002
Ethnicity (Hispanic)	−0.83	0.17	0.44	0.31	0.61	0.000
Marital Status (Married)	−0.46	0.06	0.63	0.56	0.71	0.000
Gender (Male)	0.56	0.06	1.75	1.55	1.97	0.000
Age (16–17)	0.10	0.06	1.11	0.99	1.25	0.083
Educational Attainment	−0.16	0.03	0.85	0.80	0.91	0.000
Race × Educational Attainment	0.20	0.07	1.22	1.08	1.39	0.002
Ethnicity × Educational Attainment	0.25	0.06	1.29	1.14	1.45	0.000
Constant	−0.90	0.12				0.000

Notes: Source Population Assessment of Tobacco and Health (PATH; 2013–2014). CI: Confidence interval; SE: Standard error; OR: Odds ratio.

## Appendix A

**Table A1:** Distribution of Chronic Medical Conditions (CMCs) by race, ethnicity, and age.

			12–15					16–17		
	Race		Ethnicity		All	Race		Ethnicity		All
Number of CMCs	White	Black	Non-Hispanic	Hispanic	\	White	Black	Non-Hispanic	Hispanic	
0	53.8	50.0	52.5	55.3	53.0	52.0	51.6	55.3	57.7	52.0
1	33.0	35.8	34.4	31.2	33.1	33.8	32.9	31.2	29.7	32.7
2	10.9	11.1	10.9	11.1	11.0	11.0	12.2	11.1	9.5	11.2
3	2.0	2.5	2.0	2.1	2.1	2.5	2.6	2.1	2.5	2.5
4	0.2	0.4	0.2	0.2	0.2	0.6	0.7	0.2	0.4	0.6
5	0.0	0.2	0.0	0.1	0.1	0.0	0.0	0.1	0.2	0.1
6	0.0	0.0	0.0	0.0	0.0	0.0	0.0	0.0	0/0	0.0
